# Effect Analysis of Different Environmental Disinfection Methods on Reducing Contamination of Surfaces by the Omicron BA.2.2 Variant of SARS-CoV-2 and the Characteristics of Fomite Contamination in the Fever Clinic in the Out-Broken of Shanghai

**DOI:** 10.1007/s12560-022-09545-w

**Published:** 2023-04-17

**Authors:** Lei Yang, Wenfang Dong, Xiaoyu Shen, Feng Han, Chenxi Liu, Yirou Wang, Xuemei Xu, Yufen Wu, Sha Zhou, Qing Cao

**Affiliations:** 1 Department of Cardiology, Shanghai Children’s Medical Center, School of Medicine, Shanghai Jiao Tong University, Shanghai, 200127 China; 2 Department of Respiratory Medicine, Shanghai Children’s Medical Center, School of Medicine, Shanghai Jiao Tong University, Shanghai, 200127 China; 3 Department of Pediatrics, Shanghai Children’s Medical Center, School of Medicine, Shanghai Jiao Tong University, Shanghai, 200127 China; 4 Department of Neurology, Shanghai Children’s Medical Center, School of Medicine, Shanghai Jiao Tong University, Shanghai, 200127 China; 5 Department of Rheumatology and Immunology, Shanghai Children’s Medical Center, School of Medicine, Shanghai Jiao Tong University, Shanghai, 200127 China; 6 Department of Endocrinology and Metabolism, Shanghai Children’s Medical Center, School of Medicine, Shanghai Jiao Tong University, Shanghai, 200127 China; 7 Department of Outpatient and Emergency, Shanghai Children’s Medical Center, School of Medicine, Shanghai Jiao Tong University, Shanghai, 200127 China; 8 Administration Department of Nosocomial Infection, Shanghai Children’s Medical Center, School of Medicine, Shanghai Jiao Tong University, Shanghai, 200127 China; 9 Department of Infectious Disease, Shanghai Children’s Medical Center, School of Medicine, Shanghai Jiao Tong University, Shanghai, 200127 China

**Keywords:** Fever clinic, SARS-CoV-2, Variant of Omicron BA.2.2, Contamination surfaces, Environmental disinfection methods

## Abstract

This study aimed to investigate the effect of different environmental disinfection methods on reducing contaminated surfaces (CSs) by the Omicron BA.2.2 variant of SARS-CoV-2 in the fever clinic between March 20 and May 30, 2022, and to analyze the influences and related factors of CSs. This study includes survey data from 389 positive patients (SPPs) and 204 CSs in the fever clinic, including the CS type, disinfection method, length of time spent in the clinic, cycle threshold (CT) value, name, age, weight, mask type, mask-wearing compliance, hand-mouth touch frequency and sex. Associations between study variables and specified outcomes were explored using univariate regression analyses. Mask-wearing compliance had a significant negative correlation with CSs (*r* = − 0.446, *P* = 0.037). Among the 389 SPPs, 22 SPPs (CRP, 5.66%) caused CSs in the separate isolation room. A total of 219 SPPs (56.30%) were male. The mean age of SPPs was 4.34 ± 3.92 years old, and the mean CT value was 12.44 ± 5.11. In total, 9952 samples with exposure history were taken, including 204 (2.05%) CSs. Among the CSs, the positive rate of flat surfaces was the highest in public areas (2.52%) and separate isolation rooms (4.75%). Disinfection methods of ultraviolet radiation + chemical irradiation significantly reduced the CSs in both the public area (0% vs. 4.56%) and the separate isolation room (0.76% vs. 2.64%) compared with the chemical method alone (*P* < 0.05). Compared with ordinary SPPs, CRPs were older (6.04 year vs. 4.23 year), and the male proportion was higher (72.73% vs. 55.31%). In particular, it was found that SPPs contaminated their surroundings and therefore imposed risks on other people. Environmental disinfection with ultraviolet radiation + chemical treatment should be emphasized. The findings may be useful to guide infection control practices for the Omicron BA.2.2 variant of SARS-CoV-2.

## Introduction

As the highly infectious severe acute respiratory syndrome coronavirus 2 (SARS-CoV-2) rages in parts of China, an outbreak of this pandemic, which has brought great challenges, occurred in Shanghai, the country’s financial hub and the home of many of its top research institutions, from early March to late May, 2022 (Cao et al., 2020; Kawaoka et al., 2022). Phylogenetic features of SARS-CoV-2 viral genomes collected from 129 patients in this period, comparing their relationship with those available in the Global Initiative of Sharing All Influenza Data (GISAID) database, indicated that all of the new viral genomes in Shanghai were clustered into the Omicron BA.2.2 variant, which has a near 90% likelihood of being unaffected by current vaccines (Zhang et al., 2022).

At present, the theory that the Omicron BA.2.2 variant can remain alive on the surface of a touched object (contaminated surfaces, CSs) and has a human-material-human (H-M-H) communication model is still in dispute (Chen et al., 2022). Varvara A. Mouchtouri reported that COVID-19 can be transmitted directly through respiratory droplets or indirectly through fomites. SARS-CoV-2 can be detected on various environmental surfaces and in air samples and sewage in hospital and community settings (Mouchtouri et al., 2020). M D’accolti reported that the existence of SARS-CoV-2 on hospital surfaces may be limited, and effective transmission of SARS-CoV-2 by surfaces/fomites within the hospital ward may be a rare event (D'accolti et al., 2020). Based on the above studies, it is important to analyze the characteristics of fomite contamination environments, especially in medical settings, and find more effective disinfection methods to relieve H-M-H transmission. Here, we present an effect analysis of different environmental disinfection methods for the Omicron BA.2.2 variant and the characteristics of fomite contamination in the fever clinic in Shanghai Children’s Medical Center affiliated with the Medical College of Shanghai Jiao Tong University.

## Methods

### Patient Demographics

**Fig. 1:**
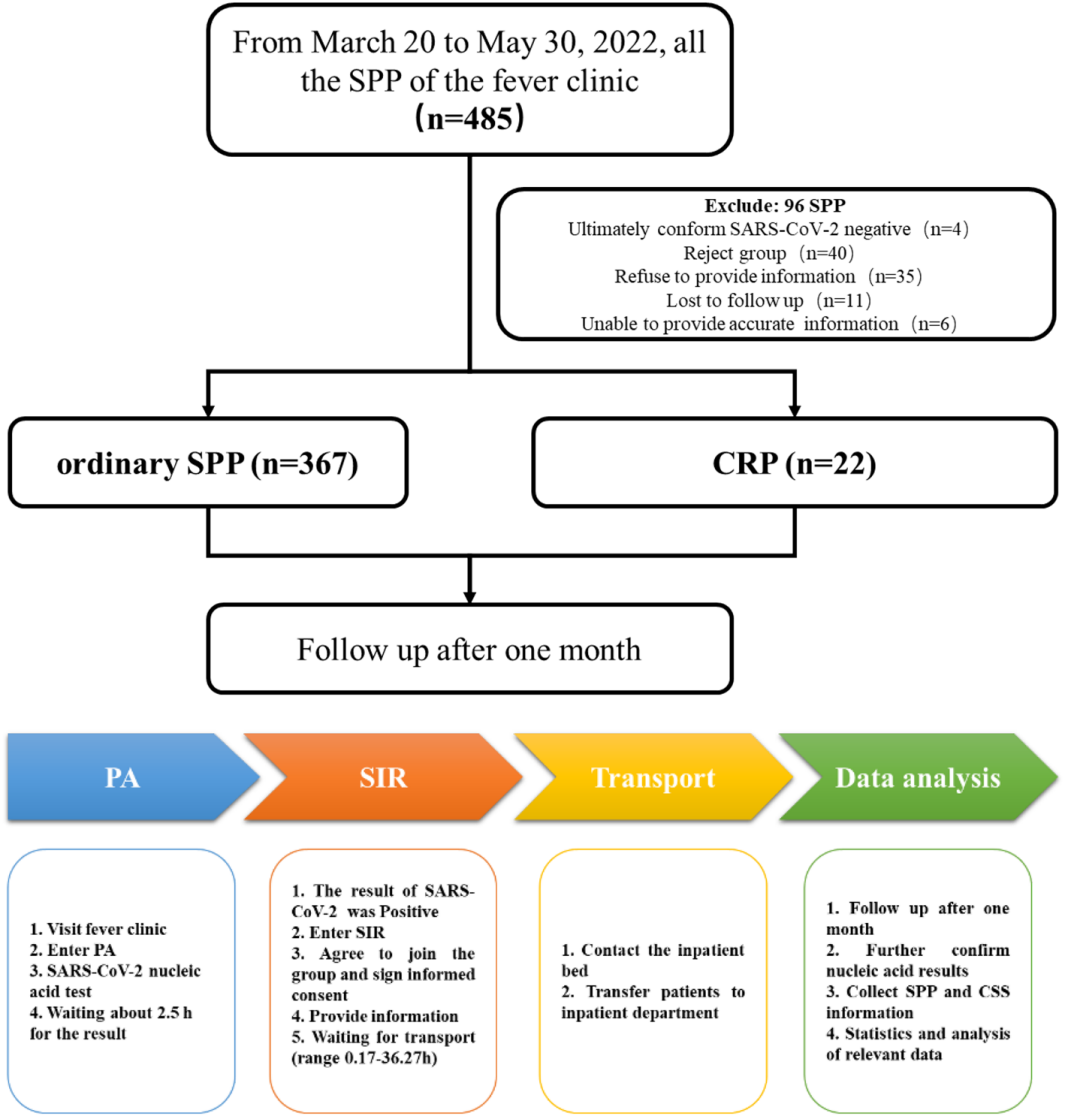
Overall design of the study. *SPP* Omicron BA.2.2 variant of SARS-CoV-2-positive patient; *CRP* Omicron BA.2.2 variant of SARS-CoV-2-positive patient who caused contaminated surfaces; *PA* public area; *SIR* separate isolation room

Data from March 20 to May 30, 2022, about CSs and the Omicron BA.2.2 variant in SARS-CoV-2-positive patients (SPPs) of the fever clinic in Shanghai Children’s Medical Center affiliated with the Medical College of Shanghai Jiao Tong University were reviewed, including the CS type, disinfection method, length of time spent in the clinic, cycle threshold (CT) value, name, age, weight, mask type, mask-wearing compliance, hand-mouth touch frequency and sex (Fig. 1).

### Ethics

Parents and/or legal guardians signed written informed consent. All data collection and analysis were approved by the local research ethics and clinical governance bodies.

Disinfection Method (used in the vacant environment after SPPs were transferred).

Chemical disinfection (March 20 to April 13, 2022): The environment was cleaned first, and then 3000 mg of trichloroisocyanuric acid disinfection tablets (Lionser company) was dissolved in 3 L of deionized water (25 ± 2 °C) for 5 min. After being completely dissolved and evenly mixed, the solution was sprayed to disinfect the surfaces. The solution was allowed to sit on the surfaces for half an hour. Finally, the surfaces of the object were wiped with disinfection wipes. Using this method, the fever clinic was disinfected 2 times a day at 5:00 and 17:00.

Ultraviolet radiation + chemical disinfection (April 14 to May 30, 2022): The environment was cleaned first and then subjected to ultraviolet irradiation for 1 h (Shanghai Yuejin nonmagnetic ultraviolet disinfection vehicle ZXC-ii). The subsequent process was consistent with the chemical disinfection method described above.

### Surface Sampling Method

A 5 cm*5 cm sterilization specification plate was placed on the surface of the tested object. A cotton swab soaked with sterile 0.03 mol/L phosphate buffer solution or normal saline sampling solution was applied horizontally and vertically to the specification plate 5 times, and then the cotton swab was rotated to continuously sample the area of the 4 specification plates. If the surface to be sampled is less than 100 cm^2^, the entire surface was swabbed; if the surface was > 100 cm^2^, only an area of 100 cm^2^ was swabbed. The part of the swab that contacted the hand was cut off, and the cotton swab was placed into a test tube containing 10 ml of eluent for the sterility test. For small objects such as door handles, the cotton swab was directly applied to the surface of the object for sampling.

### Viral Nuclei Acid Test

Real time fluorescent quantitative reverse transcription-polymerase chain reaction (RT-PCR) is the main diagnostic method of SARS-CoV-2 in clinical practice. Its basic principle is to monitor the growth of the product quantity in real time through the fluorescent labeled-specific probe, tracking the PCR product labeling in the reaction process, and calculating the initial template quantity according to the amplification curve. The SARS-CoV-2 genome contains the 5 'end replicase coding gene [open reading frame 1ab, ORF1ab] and structural protein coding gene [spike protein, S, envelope protein, E, membrane protein, M, and nucleocapsid protein, N]. Among them, ORF1ab fragment and N gene are genus and type specific genes of SARS-CoV-2. A positive case requires that the same sample be positive for both targets.

### Categories of Object Surfaces

All the object surfaces were divided into three categories, namely, flat surface (FS, surface area ≥ 100 square centimeters and low-frequency contact), hand high-frequency contact surface (HHF, surface area < 100 square centimeters and high-frequency contact) and electronic products (Eps). See Appendix 1 for specific classification contents.

### Statistical Analysis

SPSS 20.0 statistical software was used for analysis. The measurement data are expressed as the mean ± standard deviation; the counting data are expressed as the rate. One-way ANOVA was used for comparison of the means within the group, and the counting data were compared by the chi square test. Single-factor analysis was included to further examine the independent influencing factors of CSs with SPPs. All statistics were tested using a two-sided test, and *P* < 0.05 was considered statistically significant.

## Results

### Patient Demographics

**Fig. 2:**
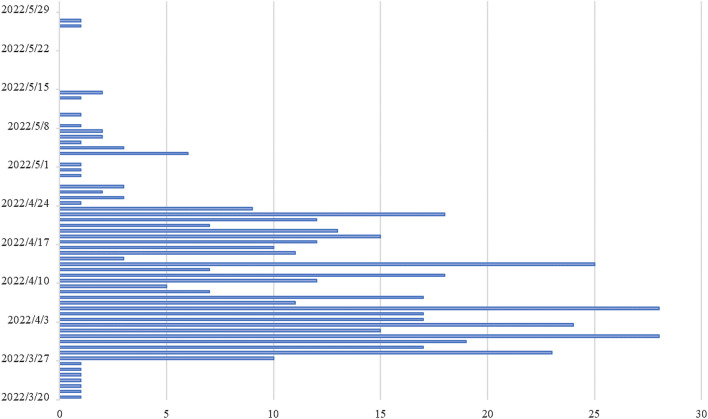
The daily number of SPPs

From March 20 to May 30, 2022, a total of 485 SPPs went to the fever clinic, of which 389 were finally included in the study, and 22 SPPs (5.66%) caused CSs in the separate isolation room. A total of 219 SPPs (56.30%) were male. The mean age of SPPs was 4.34 ± 3.92 years old, and the mean CT value was 12.44 ± 5.11. In total, 9952 samples with exposure history (SPPs in the fever clinic on that day or in the separate isolation room) were taken, including 204 (2.05%) CSs (Table 1, Fig. 2).

**Table 1:** Patient Demographics

Characteristics of SPP	Mean ± standard deviation	Range
Gender: Male	219	
Age (year)	4.34 ± 3.92	0.04–16
Weight (kg)	20.32 ± 13.67	3.4–75
Height (cm)	102.60 ± 30.60	51–178
CT value	12.44 ± 5.11	6–37.79
Duration of time in clinic (h)	11.55 ± 8.63	0.17–36.27
N95 mask-wearing	30	

*SPP* Omicron BA.2.2 variant of SARS-CoV-2-positive patient

### Univariate Analysis Model Identifies Factors Related to CSs

The results of the analysis of variables associated with CSs are presented in Table 4. The analysis confirmed that the daily number of SPPs was positively associated with CSs (*r* = 0.358; 95% CI [0.050–0.692]). The CT value (*r* = − 0.38; 95% CI [− 0.507–0.523]) and mask-wearing compliance (*r* = − 0.446; 95% CI [− 0.698–0.043]) were both negatively associated with CSs; however, only mask-wearing compliance was statistically significant (Table 2).

**Table 2:** Univariate analysis model identifies the factors related to CSs

Variable	Coefficient (*r*)	*P* value	95% CI
Number of SPPs daily	0.358	0.079	0.050–0.692
Duration of time in clinic (h)	− 0.244	0.329	− 0.641–0.238
Age (Year)	− 0.015	0.951	− 0.511–0.465
Body surface area (m^2^)	− 0.031	0.904	− 0.466–0.370
CT value	− 0.38	0.882	− 0.507–0.523
Mask-wearing compliance	− 0.446	0.037	− 0.698–0.043
Hand-mouth touch frequency	0.136	0.546	− 0.194–0.501

### Comparison of Different Types of CSs

From March 20 to May 30, 2022, the number (rate) of CSs in the FS, HHF and EP groups was 40 (2.52%), 22 (1.89%) and 11 (1.73%), respectively, in the PA and 87 (4.75%), 65 (3.00%) and 35 (1.82%) in the SIR. Among the three categories, FS had the highest positive rate, and EP had the lowest. However, there was no significant difference among the three groups (*P* > 0.05) (Table 3).

**Table 3:** Comparison of different types of CSs in public areas and separate isolation rooms

CSs	Public area (rate)	Separate isolation room (rate)
Total	73 (2.15%)	131 (2%)
Chemical disinfection	73 (4.56%)	114 (2.64%)
Ultraviolet radiation + chemical disinfection	0 (0.00%)*	17 (0.76%)*
Flat surface	40 (2.52%)	87 (4.75%)
Hand high-frequency contact surface	22 (1.89%)	65 (3.00%)
Electronic product	11 (1.73%)	35 (1.82%)

*Compared with chemical disinfection, *P* < 0.05

*CSS* contaminated surfaces by the Omicron BA.2.2 variant of SARS-CoV-2

### Comparison of CSs with Different Disinfection Methods

**Fig. 3:**
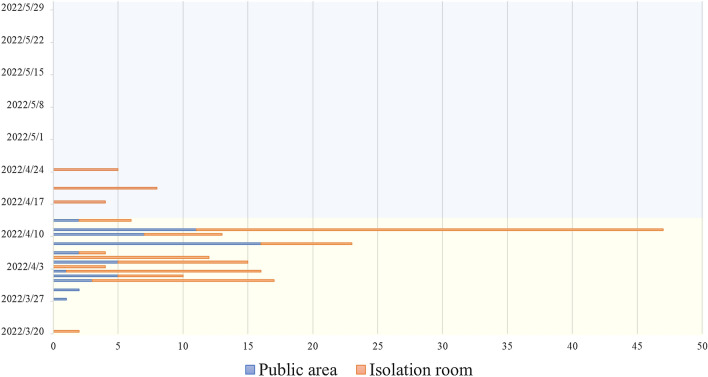
The daily number of CSs. *Note*: The blue line represents CSs in the public area, the orange line represents CSs in the separate isolation room, the canary yellow background represents chemical disinfection alone, and the light blue background represents ultraviolet radiation + chemical disinfection

In the fever clinic, patients need to have a SARS-CoV-2 nucleic acid test first in the public area (PA), and the waiting time for the results is approximately 2.5 h. If positive, they will enter a separate isolation room (SIR) with a parent for separate isolation and treatment, waiting for transfer. Before the statistical analysis, negative samples with no exposure history (no SARS-CoV-2-positive children in the fever clinic on that day or in that SIR) were excluded. From March 20 to May 30, 2022, the number (rate) of CSs in the PA and SIR was 73 (2.15%) and 131 (2%), respectively. When using the chemical disinfection method, the number (rate) of CSs in the PA and SIR was 73 (4.56%) and 114 (2.64%), respectively. After using ultraviolet radiation + chemical irradiation, the CSs were 0 (0%) and 17 (0.76%), which both decreased significantly (*P* < 0.05) (Table 3, Fig. 3).

### Comparison of Characteristics of the Ordinary SARS-CoV-2 Patients and Contaminated Surface-Related SARS-CoV-2 Patients

Patients who caused CSs were defined as having contaminated surface-related SARS-CoV-2 patients (CRPs) and all other patients were defined as ordinary SARS-CoV-2-positive patients (OSPPs). The male proportion, age, weight, height, CT value, N95 mask-wearing rate and time spent in the room were 55.31%, 4.23 ± 3.89 years, 20.08 ± 13.72 kg, 101.85 ± 30.64 cm, 12.44 ± 5.15, 7.63% and 16.46 ± 4.65 h, respectively, in OSPPs and 72.73%, 6.04 ± 4.04 years, 24.38 ± 12.36 kg, 115.14 ± 27.67 cm, 12.39 ± 4.52, 9.09% and 14.52 ± 9.38 h in CRPs. Compared with OSPPs, CRPs were older and the male proportion was higher, but none of the differences were significant (Table 4).

**Table 4:** Comparison of the characteristics of ordinary SARS-CoV-2-positive patients and contaminated surface-related SARS-CoV-2 patients

Characteristics of SPP	OSPP	CRP
Total	367	22 (5.66%)
Gender: Male	203 (55.31%)	16 (72.73%)
Age (Year)	4.23 ± 3.89	6.04 ± 4.04
Weight (kg)	20.08 ± 13.72	24.38 ± 12.36
Height (cm)	101.85 ± 30.64	115.14 ± 27.67
CT value	12.44 ± 5.15	12.39 ± 4.52
N95 mask-wearing	28 (7.63%)	2 (9.09%)
Duration of time in clinic (h)	16.46 ± 4.65	14.52 ± 9.38

*OSPP* ordinary SARS-CoV-2-positive patients who did not cause CSs, *CRP* contaminated surface-related SARS-CoV-2 patients who did cause CSs

## Discussion

In early March 2022, a wave of SARS-CoV-2 infection rapidly appeared in Shanghai, China, and the phylogenetic features indicated that all of the new viral genomes were clustered into the Omicron BA.2.2 (Zhang et al., 2022) variant. With the development of the epidemic, a new fever clinic was established and put into operation in our hospital on March 18. On March 20, the first SPP entered the fever clinic, and as of May 30, when the epidemic had essentially ended, 485 SPPs had been accepted by the fever clinic. During this period, 9952 samples with a history of exposure were taken, with a total of 204 CSs, accounting for 2.05%. Among the CSs, the positive rate of flat surfaces was the highest in both the PA and SIR. The disinfection method of ultraviolet radiation + chemical irradiation effectively reduced CSs in both the PA and SIR. Compared with OSPPs, CRPs were older, and the proportion of males was higher.

The Omicron BA.2.2 variant has extremely high infectivity. It is approximately 1.5 and 4.2 times as contagious as BA.1 and Delta, respectively, due to four unique mutations in the receptor-binding domain and 12 shared with BA.1 (Kawaoka et al., 2022). In contrast, the Delta variant has only two receptor-binding domain mutations (Farinholt et al., 2021). Furthermore, nationwide Danish data in late December 2021 and early January 2022 indicate that the BA.2 variant is inherently substantially more transmissible than BA.1 and capable of vaccine breakthrough (Espenhain et al., 2021). If no comprehensive understanding of CSs, CRPs and strict disinfection methods were undertaken, the number of severe to critical cases and the resultant death toll could be high in Shanghai, with a population of 25 million, a similar situation to that reported in Hong Kong (Gu et al., 2022).

The contamination of the Omicron BA.2.2 variant on the surface of objects, that is, CSs, leads to the H-M-H communication mode increasing the SARS-CoV-2 infection rate, which has attracted increasing attention. However, at present, there are few studies in this area, and the existing research conclusions also have many contradictions. Li Wei et al. confirmed extensive contamination of SARS-CoV-2 patients' surroundings, therefore imposing risks for other people (Wei et al., xxxx). Perrine Marcenac et al. question whether the virus can truly spread first through surfaces (Marcenac et al., 2021). In our study, it was found that there were a large number of CSs in the environment. Because the Omicron BA.2.2 variant is highly infectious, the risk of transmission is noteworthy. Therefore, we must recognize the importance of CSs in SARS-CoV-2 transmission and actively intervene and block the H-M-H transmission mode to reduce the infection rate of SARS-CoV-2.

In our study, it was found that the more SPPs who visited the clinic, the more CSs there were. However, due to the existence of CRPs, the risk is not small if the number of SPPs is small. For example, on March 23, there was only one SPP in the fever clinic, but 11 CSs were caused by that patient. Daily disinfection must be carried out strictly regardless of the number of SPPs.

It has been reported that the chemical disinfection which can oxidize SARS-CoV-2 mercapto group, interact with cytoplasmic components to form nitrogen-chloride complex, interfering with virus metabolism (Viana Martins et al., 2022; Xiling et al., 2021) or ultraviolet radiation which can destroy the molecular structure of SARS-CoV-2 RNA, causing viral growth and replication barriers alone (Heilingloh et al., 2020; Viana Martins et al., 2022) is effective against SARS-CoV-2 by referring to previous laboratory and clinical studies. However, in the real world, especially when facing such a high exposure intensity as the Shanghai pandemic, the chemical method alone is obviously not enough for the Omicron BA.2.2 variant. In our research, it was found that the number of CSs after chemical disinfection alone was still high for both the PA and SIR but decreased significantly with the use of the ultraviolet radiation + chemical method, showing that ultraviolet radiation + chemical disinfection is necessary to eliminate SARS-CoV-2 in the environment, especially in medical places.

According to the different characteristics of object surfaces in the fever clinic, they were divided into three categories, namely, flat surfaces (with low-frequency hand contact), high-frequency hand contact surfaces and electronic products (also with high-frequency hand contact). Among the three categories, flat surfaces had the highest positive rate, and electronic products had the lowest positive rate, meaning that a greater surface area rather than the amount of hand contact may affect the risk of transmission in indoor environments.

Due to the design of consulting procedures, the Omicron BA.2.2 variant exposure modes in the PA and the SIR are not exactly the same. The PA is open and well ventilated, and each SPP enters while the SIR is separately closed, and each SIR is only accessed by one SPP. Previous studies on the environment of SARS-CoV-2 mainly focused on the confined space, and the exposure mode was similar to the SIR, while there were few studies on the PA, and there were different opinions on the CSs of the confined space (Marcenac et al., 2021; Viana Martins et al., 2022). In this study, it is believed that SARS-CoV-2 can be detected in the home environment of patients with COVID-19, but the risk of transmission through pollutants is very low (Marcenac et al., 2021). Christopher Bartlett et al. suggested that SARS-CoV-2 can be transmitted through pollutants in the confined space of the hospital (Bartlett et al., xxxx). In this study, the positive rate of CSs was similar in the PA and the SIR, which indicates that the Omicron BA.2.2 variant has strong adhesion. Whether SARS-CoV-2 patients stay in the PA or the SIR, the surfaces are polluted, so attention should be given to environmental disinfection.

We are aware of limitations in this study. The pandemic lasted only slightly more than two months, and the amount of dataare limited. We tested only for viral nucleic acids and did not perform viral culture to test viability. Despite the limitations, we believe that the findings reported here may help to guide the prevention and control of SARS-CoV-2.

## Conclusion

The surroundings of SPPs can be extensively contaminated, imposing risks for others in contact with them due to CSs. Disinfection with ultraviolet radiation + chemical can effectively reduce the risk compared with chemical disinfection alone (overall efficiency 99.58% vs 96.84%).

## Appendix

See Table 5

**Table 5:** Specific CSS classification contents

Location	Flat surface	Electronic product	Hand contact with high-frequency
Isolation room (10)	Bed surface, chair surface, consulting room tabletop, garbage can surface, wash basin surface, computer screen	Keyboard, telephone button, mouse, handset handle	Chair armrest, inner/outer door handle, faucet, switch, sterilizer
Hall	Chair surface, hall machine surface		Machine card reader, report exit, chair armrest
Infusion room	Chair surface, bar counter		Inner/outer door handle, infusion rod
Pre inspection platform	Tabletop, chair surface, screen	Mouse, keyboard, card reader	Chair armrest, sterilizer
Nucleic acid sampling	Window, tabletop, side wall, door curtain		Sterilizer, bar code machine, inner/outer door handle
Toilets	3 faucets, sterilizer Wash basin, 3 countertops, garbage can surface, mirror		
Laboratory	Windows, countertops, screens, seats	Bar code machine, reporting machine, card reader	Sterilizer
Registration office	Window, table, screen	Credit card machine	Sterilizer
Pharmacy	Window, table, screen	Credit card machine	Sterilizer
Nurse station	Screen	Keyboard, mouse, telephone keys, handset handle	Sterilizer
Baby care room	Table, chair		Inner/outer door handle and window handle
Emergency room	Bed surface, chair surface, consulting room tabletop, garbage can surface, wash basin surface, computer screen	Keyboard, telephone button, mouse, handset handle	Chair armrest, inner/outer door handle, faucet, switch, sterilizer
